# Beyond digital interfaces: The human element in online teaching and its influence on student experiences

**DOI:** 10.1371/journal.pone.0307262

**Published:** 2024-07-31

**Authors:** Soo See Chai, Su-Hie Ting, Kok Luong Goh, Yee Hui Robin Chang, Bui Lin Wee, Dila Novita, J. Karthikeyan

**Affiliations:** 1 Faculty of Computer Science and Information Technology, University of Malaysia Sarawak (UNIMAS), Kota Samarahan, Sarawak, Malaysia; 2 Faculty of Language and Communication, University of Malaysia Sarawak (UNIMAS), Kota Samarahan, Sarawak, Malaysia; 3 School of Science and Technology, International University College of Advanced Technology Sarawak (iCATS University College), Kuching, Sarawak, Malaysia; 4 Faculty of Applied Sciences, Universiti Teknologi MARA Cawangan Sarawak, Jalan Meranek, Kota Samarahan, Sarawak, Malaysia; 5 Department of Public Administration, Universitas Islam 45, Bekasi, Indonesia; 6 National College (Autonomous)Tiruchirappalli, Tiruchirappalli, Tamilnadu, India; Ataturk University, Faculty of Pharmacy, TÜRKIYE

## Abstract

Amidst the digital transformation of education, the essence of the human touch in online teaching remains pivotal. Despite growing literature, there remains a significant gap in understanding how the human element in online teaching directly influences student engagement and learning outcomes, especially in diverse educational contexts. This study develops a quantifiable index capturing the essence of humanized online teaching and investigates the determinants influencing this humanization. Additionally, an index encapsulating students’ online learning experiences, as perceived by their instructors, has been constructed. Bridging these indices, the research unravels the intricate relationship between the humanization of online teaching and the resulting student experiences in the virtual realm. Sourced from a self-constructed questionnaire and encompassing responses from 152 instructors across 22 Malaysian institutions, the data revealed an average incorporation of 81.38% humanized online teaching elements. Key determinants, such as subject matter, teaching experience, Internet quality, and platform choices, emerged as significant influences. A regression model showed approximately 31.7% (R-squared = 0.317, p<0.001) of the variation in the dependent variable. A significant moderate positive correlation (r = 0.423, p<0.001) between the Humanized Online Teaching Index and the Students’ Online Learning Experiences Index highlights the intertwined nature of humanized instructional methodologies and enhanced student engagement in online settings. Though contextualised during the coronavirus disease-2019 pandemic, the study’s implications transcend the immediate circumstances, offering transformative insights for future online teaching methodologies and enhancing student experiences in the evolving digital age.

## Introduction

Distance education has been around for 300 years [1], but today’s distance learning utilizes the Internet to facilitate real-time communication between teachers and students. When the COVID-19 pandemic in 2020 resulted in a widespread shift to online learning, video conferencing platforms such as WebEx, Zoom, and Google Classroom were being used to help students learn despite school closures. Online learning, or e-learning, involves using electronic devices connected to the Internet to learn in either synchronous or asynchronous settings. It is a type of remote learning that bridges the physical and geographical distances between teachers and students, fostering an accessible educational environment irrespective of their locations [2]. If not utilized properly, the use of technology can potentially hinder learning as it may remove its social component and cause students to feel isolated. This sense of isolation can lead students to perceive themselves as just users of certain software rather than being part of an educational institute [3], which can negatively affect their engagement, motivation, retention, and satisfaction [4].

Thus, online education must leverage technology while preserving a human touch to reduce isolation [5]. The human touch is an integral aspect of conventional classroom teaching, whereby instructors and students can interact, laugh together, and make learning an enjoyable experience [6]. Humanizing instruction relies on instructor-student interactions, which result in a connection between students, engagement, and rigorous exchange [7]. Humanizing instruction involves creating opportunities for such interactions and fostering a caring environment [8]. When done in the course of online learning where screen is the medium of instruction, maintaining human interactions presents a challenge. Some teachers suggest using the camera-on mode to establish a human touch in online learning [9]. With the camera off, the absence of visual cues makes it difficult for teachers to know whether students can understand the teachings. However, mandating students to turn on their cameras may not be culturally sensitive or equitable [10].

The four interconnected elements that underpin humanized online instruction are: Trust, Presence, Awareness, and Empathy [11, 12]. These principles of humanization in online teaching are closely related to the Community of Inquiry (CoI) framework, which we use in our study to investigate students’ online learning experiences from their instructors’ perspectives. CoI is a widely recognized theoretical framework that provides a holistic view of the online learning experience, emphasizing the importance of social, cognitive, and teaching presence [13]. The CoI framework posits that effective online learning, particularly higher-order learning, requires community building [14]. Integral to our study, it offers a comprehensive model for analyzing the dynamics of online learning environments. The social presence in the CoI framework refers to the ability of participants to project their personal characteristics into the community, thereby supporting communication and relationship building [15]. Cognitive presence, the core of the CoI model, involves the exploration, construction, resolution, and confirmation of understanding through collaboration and reflection [16]. Teaching presence, the design and facilitation aspect of the framework, entails orchestrating the other two presences to support learning [17]. Research has consistently shown that these interdependent elements of the CoI framework are essential for creating a meaningful and holistic online learning experience, emphasizing the importance of integrating social, cognitive, and teaching strategies for effective online education [18]. By examining how instructors integrate humanizing approaches into their online instruction, we can better understand how to create a sense of community and promote active learning among online learners, which are essential components of the CoI framework. In this context, the primary challenge is to ensure how, even without enforcing camera-on mode during class time, instructors may integrate human touch in the learning process through a variety of approaches.

In the evolving landscape of education, the humanization of online teaching has emerged as a pivotal factor in enhancing the quality of virtual learning experiences. This research employed the three interdependent elements of the CoI framework to determine if students are enjoying profound and meaningful learning experiences from the perspective of their instructors. Specifically, our study investigates how extensively instructors at Malaysian higher education institutes have integrated humanization principles into their online teaching, which aligns with the social presence component of the CoI framework. Although there has been considerable research on the subject of humanizing online courses using various methods, little academic attention has been paid to what extent the four principles of humanization in online teaching have been incorporated into online classes.

This study embarked on a two-fold mission: first, to develop a quantifiable index that captures the degree to which instructors’ online teaching is humanized; and second, to delve into the determinants that influence this humanization. The reliability and internal consistency of the questions used in the survey for examining the extent to which online teaching has been humanized, measured by Cronbach’s alpha [19], was the first step in developing the index. Furthermore, we constructed another index that encapsulates students’ online learning experiences as perceived by their instructors. By bridging these two indices, our research sought to unravel the intricate relationship between the humanization of online teaching and the resulting student experiences in the virtual learning environment. This research sheds light on the symbiotic interplay between instructional methodologies and student engagement, offering insights that could redefine the paradigms of online education.

In the subsequent sections, this paper systematically unfolds its narrative. Starting with the Method section, we detail our research methodology, encompassing sample selection, questionnaire development, and data collection. This is complemented by the Development of the Questionnaire section, where the process of creating a bespoke tool to assess humanized online learning and the CoI framework is elucidated. The Measures section then delves into the specifics of the questionnaire, followed by Statistical Analysis, where we describe our analytical techniques. The Results section presents pivotal findings, including insights into participant demographics, technological usage in teaching, and the infusion of the human touch in online education. Subsequently, the Discussion section contextualizes these findings within the wider educational landscape, drawing connections to the broader implications of online teaching. The paper concludes by summarizing key takeaways and their broader impact, while acknowledging the study’s limitations and suggesting avenues for future research. The structure of this paper offers a comprehensive, interconnected exploration of the humanization of online teaching, its measurement, and its influence on the educational experience.

## Method

### Sample and procedure

A total of 152 educators were recruited using purposive sampling from 22 higher education institutes in Malaysia. The researchers invited their colleagues to participate via email and social media platforms, and enlisted the help of peers from other colleges and universities to distribute the online questionnaire. Data collection took place from January 12, 2022 to February 24, 2022.

We used a self-constructed questionnaire as there were no similar measures of human touch in online learning when this research was being conducted. The self-constructed online questionnaire was designed using Google Forms. The questionnaire opened with details about the purpose of the study, participants’ voluntary participation and anonymity, and researchers’ contact information. Before beginning the survey, participants were required to give their consent by signing a consent form.

The research was granted ethical approval (HREC(NM)/2020(1)/04) by the Research Ethics (Non-Medical) Committee at the University of Malaysia Sarawak (UNIMAS).

### Development of the questionnaire

In addressing the principles of humanized online learning and the CoI framework, this study acknowledges the gap in current research regarding specific measurement tools. Though existing literature extensively discusses the theoretical aspects of humanizing online education and the CoI framework, there is a notable absence of comprehensive questionnaires designed to quantitatively assess these constructs in practical educational settings. Existing studies primarily focus on qualitative analyses or general discussions of the principles involved, such as trust, presence, awareness, empathy, and the CoI’s three presences (social, cognitive, and teaching) [11–14]. Recognizing this gap, our research contributes to the literature by developing and implementing a specific questionnaire aimed at evaluating these principles in a structured manner. This original questionnaire is derived from the theoretical underpinnings of the aforementioned frameworks, but tailored to capture the nuanced realities of online teaching and learning experiences. It is an advancement in the field by as a tool for empirical investigation into the practical application of these widely acknowledged educational theories.

### Measures

Table 1 provides a summary of the elements of the four principles of humanized online learning, as well as the questionnaire items used to evaluate the principles of humanized online learning in this study. Table 2 provides an overview of the design elements for each of the three presences in the CoI framework, based on which the questionnaire items were constructed.

**Table 1:** Questionnaire items based on the principles of humanized online learning.

Principle	Elements	Questionnaire Item		
		Question (No.)	μ	*σ*
Trust	• Honest and transparent communication • Illustrating “humanness” with selective snapshots of your life via storytelling, pictures, or experiences	How often do you contact students before or after online class to add a human touch to your teaching? (Q16)	5.18	1.11
		How often do you post/show pictures of yourself so that students get to know you? (Q18)	5.26	1.58
		How often do you share your personal experiences during online teaching? (Q22)	4.76	1.72
Presence	• Provide frequent text, audio, or video presence • Verbal immediacy includes the use of humor, encouragement, and use of student names • Encourage student-to-student interaction	How often do you ask students to post/show a photo of themselves in order to put a face with a name or show their faces via camera? (Q17)	**3.41**	**2.13**
		How often do you reply to students’ comments in chat/discussion threads to give them individual attention? (Q20)	**5.81**	**0.60**
		How often do you use your students’ names during online class to make them feel they are truly individuals? (Q21)	5.72	0.93
		How often do you provide opportunities/activities for students to make friends with one another? (Q23)	4.47	1.76
		How often do you use humor (e.g., make jokes) in online class? (Q27)	5.10	1.45
Awareness	• Discover and get to know your students and provide comprehensive support based on your students’ needs	How often do you chit-chat with students about their interests to make a personal connection with them when having online class? (Q19)	4.80	1.62
		How often do you monitor students who are passive during online class? (E.g., contact them personally using email or social media, ask their friends to contact them) (Q26)	4.09	1.64
		How quickly do you respond to students’ questions? (Q28)	4.20	0.80
Empathy	• Understand the learner’s feelings, perceptions, challenges, and/or needs	How often do you check if your students are emotionally okay (i.e., not confused, anxious or frustrated)? (Q24)	4.78	1.60
		How often do you remind your students of assignment/homework due dates during online class? (Q25)	5.05	1.24

**Table 2:** Mapping of questionnaire questions with social, cognitive, and teaching presence of the community of inquiry framework.

Presence	Design Element	Student Experience	Questionnaire Item		
			Question (No.)	μ	*σ*
Social	Communication	Valuing of learning	My students often feel shy to ask questions in class. (Q30)	2.78	1.15
	Affective expression	Expressing emotion	My students often tell me of their personal problems that prevent them from attending classes. (Q31)	**2.44**	1.00
	Group cohesion	Opportunity to express view	My students often tell me that they have more work to do when it is online classes. (Q32)	2.75	1.10
Cognitive	Demonstrate reflection	Personal understanding	My students often tell me that they fail to understand the lessons. (Q29)	3.83	0.99
	Suggestion	Student-centered learning	I often ask my students which kind of activities they would like to have when I teach them online. (Q33)	3.40	0.97
	Challenge or question	Sense of puzzlement	I often given students problems/questions that attract them to investigate, explore more, and make them curious. (Q35)	4.10	0.69
Teaching	Design and organization	Diverse ways of learning	I often find different ways to explain concepts to students so that they can understand better. (Q34)	**4.22**	0.64
	Establish time parameters	Flexibility	I am often lenient about assignment/homework submission due dates during the COVID-19 crisis. (Q36)	4.05	0.92
	Timely feedback	Value feedback	I often encourage students to give me feedback on my online teaching after my lesson. (Q37)	3.78	0.96

The first part of the 37-item questionnaire (Section A) aimed to gather demographic information from the respondents (6 items), such as their sex, age, highest level of education, teaching experience, university, subjects taught, and their preferences toward online teaching prior to the COVID-19 pandemic. Participants’ sex, age, highest degree, and teaching experience were collected as single data points, and an ordinal scale of 1 (Do not like it at all) to 5 (Like it a great deal) was used to assess their online teaching preferences. Open-ended questions were also included in this section to inquire about the universities and courses the instructors taught.

Section B (8 items) collected data on the software and hardware technologies used before and during the pandemic, and those that the respondents anticipated they would continue using post-pandemic. The survey used single-response questions to gather data on the participants’ data plans and the type of Internet connection they used for online teaching. Multiple-response questions were used to inquire about the devices and platforms instructors used before and during the pandemic, as well as those they plan to use for online teaching post-pandemic. The questionnaire also explored the entities that assist online instructors during technological difficulties.

Section C (13 items) focused on measuring the human touch construct in online teaching with an ordinal scale ranging from 1 (Once in a semester) to 6 (Almost every class) for the first 12 items, and from 1 (Sometimes I do not respond) to 5 (Within the same hour) for the final item.

Based on the CoI framework, Section D (9 items) aimed to examine the online learning experiences of students from the perspective of their instructors, using an ordinal scale of 1 (Strongly Disagree) to 5 (Strongly Agree). There was an additional open question about how online teaching affected educators’ lives. The measurement framework for the questionnaire is tabulated in Table 3.

**Table 3:** Framework of the measurements in the questionnaire.

Item	Measurements	Scale
Q1–Q6	Section A: Demographic Background Sex, Age, Highest Qualification, Teaching Experiences Online teaching preference prior to pandemic 1–5 ordinal scale University, Subjects Taught	
		Nominal
		Ordinal
		Open Question
Q7–Q15	Section B: Use of Technology for Teaching	Nominal
	Single item data Multiple responses data	
Q16–Q28	Section C: Human Touch in Online Teaching	Ordinal
	Q16–Q27 1–6 Ordinal scale	
	Q28 1–5 Ordinal scale	
Q29–Q37	Section D: Experiences of Online Teaching	Ordinal
	1–5 Ordinal scale	
Q38	Comments	Open Question

### Statistical analysis

The data obtained were processed and analyzed using the statistical package IBM SPSS Statistics (version 28.0.1.0) for Windows.

## Results

### Demographic profile

Table 4 presents the demographic characteristics of the participants. The majority of participants are female (65.1%), and most fall between the ages of 31 and 50 (83.6%). A considerable percentage of participants have over 10 years of teaching experience (73.7%) and hold a PhD degree (52.6%). As an open-ended question was used to inquire about the subject respondents’ taught, responses were categorized into Science and Engineering (46.1%) or Art and Humanities (53.9%). Regarding online teaching preferences before the COVID-19 pandemic, 42.1% of participants were ambivalent, whereas 10.5% indicated they did not like it at all, accounting for 52.6% of the total participants. Only a small percentage of participants (5.3%) reported liking online teaching a lot before the pandemic, whereas 19.1% liked it a little, and 23.0% only somewhat liked it.

**Table 4:** Participants’ demographics profile.

Variables	Category	Count	%
Sex	Male	53	34.9
	Female	99	65.1
Age	21–30	2	1.3
	31–40	57	37.5
	41–50	70	46.1
	51–60	22	14.5
	> 60	1	0.7
Highest Educational Qualification	Diploma in education	1	0.7
	Degree (not in education)	2	1.3
	Degree in education	5	3.3
	Master’s	64	42.1
	PhD	80	52.6
Teaching Experiences	< 1 year	1	0.7
	1–3 years	10	6.6
	4–6 years	12	7.9
	7–9 years	17	11.2
	10–12 years	26	17.1
	13–15 years	28	18.4
	> = 16 years	58	38.2
University	Curtin University, Malaysia	2	1.3
	ICATS University College	6	3.9
	Kolej Komuniti Santubong	1	0.7
	Kolej Poly-Tech Mara	1	0.7
	Multimedia University	2	1.3
	University of Nottingham, Malaysia	1	0.7
	Sunway University	3	2.0
	Swinburne University of Technology, Sarawak Campus	10	6.6
	Taylor’s University	1	0.7
	UiTM	30	19.7
	UMK	1	0.7
	UMP	2	1.3
	UMS	2	1.3
	UNIMAS	75	49.3
	UNISZA	1	0.7
	UNITEN	4	2.6
	UPSI	1	0.7
	USM	3	2.0
	UTeM	1	0.7
	UTM	3	2.0
	UTS	1	0.7
	UUM	1	0.7
Subject Taught	Science and Technology	70	46.1
	Art and Humanities	82	53.9
Online Teaching Preference–Prior Pandemic	Do not like it at all	16	10.5
	Like it a little	29	19.1
	Like it a lot	8	5.3
	Neutral	64	42.1
	Somewhat like	35	23.0

### Technology used for online teaching

Table 5 provides a summary of the technologies the participants used in their online classes before, during, and after the pandemic.

**Table 5:** Technologies used before, during, and after the COVID-19 pandemic.

Variables	Category	Count	%
Data Plan	Insufficient	3	2.0
	Enough	48	31.6
	Unlimited	101	66.4
Internet Connectivity	Broadband	139	91.4
	Mobile Data	13	8.6
Devices Used During Pandemic	Smartphone	57	20.6
	iPad/pad/tablet	24	8.7
	Desktop Computer	69	24.9
	Laptop	125	45.1
	Other	2	0.7
Platforms			
i. Before Pandemic-Contact students	Email	53	31.2
	WhatsApp	88	51.8
	Facebook	5	2.9
	Telephone	15	8.8
	Other	7	4.1
	Not contacting	2	1.2
ii. Before Pandemic-Conduct Class	Microsoft PowerPoint	146	36.0
	Blackboard or Moodle	67	16.5
	Recorded lessons	19	4.7
	Online fun quizzes such as Kahoot	67	16.5
	YouTube videos	92	22.7
	Other	13	3.2
	Did not use technology	1	0.2
iii. During Pandemic- Conduct Class	Zoom	69	14.6
	Skype	4	0.9
	Webex	75	15.9
	Moodle	30	6.3
	Microsoft Teams	62	13.5
	WhatsApp	88	18.7
	Email	56	11.7
	Google Meet	62	13.0
	Other	26	5.4
iv. After Pandemic-Conduct Class (Intended)	Microsoft PowerPoint	132	21.3
	Blackboard or Moodle	63	10.2
	Recorded lessons	94	15.2
	Online quizzes such as Kahoot	86	13.9
	YouTube videos	110	17.7
	Online platforms (e.g., Zoom, Skype, Webex, Microsoft Teams, Google Meet)	122	19.7
	Other	13	2.1
Help for technical difficulties during online teaching	Nobody	100	37.9
	Family	30	11.4
	Friends	62	23.5
	Other teachers	36	13.6
	School administrative staff	32	12.1
	Other	4	1.5

### Human touch in online teaching

Table 1 displays the mean and standard deviation for the 13 items related to humanized online teaching as reported by the participants. Responding to students’ comments was the most commonly practised humanized approach (Q20: mean = 5.81). By contrast, posting or displaying a photo of themselves was the most unusual practice (Q17: mean = 3.41). Though the responses were closely clustered around the mean for Q20 (std = 0.6), there were disparities in the responses to Q17 (std = 2.13).

### Students’ experiences of online learning

Table 2 presents the mean and standard deviation of the responses obtained. Instructors were effective in teaching presence by utilizing a variety of methods to explain concepts to their students in order to facilitate better comprehension (Q34: mean = 4.22, std = 0.64). However, the instructors were generally ineffective in allowing students to vent their emotions, particularly regarding the reasons for their absence from classes (Q31: mean = 2.44, std = 1.00).

### Cronbach’s alpha

Cronbach’s alpha, or coefficient alpha, is the most commonly used statistical measure for assessing the internal reliability or consistency, and item interrelatedness of a questionnaire scale [19]. Its ideal value of acceptance is ambiguous, with the frequently cited acceptable range being 0.70 or above [20–22]. However, [23] stated that the value of Cronbach’s alpha between 0.6 to 0.8 is deemed acceptable. The value of Cronbach’s alpha is influenced by several factors, including the number of items and the interrelatedness of those items [24–26].

The alpha values obtained for each of the principles were less than 0.70 (Trust = 0.331, Presence = 0.582, Awareness = 0.517, Empathy = 0.507) (Table 6), which is mostly due to the small number of questions used for each principle. As it is impacted by the length of the test, additional test items assessing the same idea should be added to increase the value [26]. Accordingly, the reliability and consistency of all the items on humanized online learning are examined as a whole. This approach aligns with the preference for multi-item measures over single-item measures in evaluating psychological attributes or perceptions. Multi-item measures are favoured due to their ability to reduce random measurement error, enhance precision, and provide a more comprehensive representation of complex constructs [27, 28]. The Cronbach’s Alpha Based on Standardized Items was employed for Q28, which utilized a different scale compared to the other 12 items. A score of 0.782 was obtained, suggesting that all items on the test of humanized online learning contribute positively and sufficiently to the assessment of the same construct.

**Table 6:** Cronbach’s alpha for the four principles of humanized online learning.

Principle	Cronbach’s alpha
Trust	0.331
Presence	0.582
Awareness	0.517
Empathy	0.507

**Fig 1:**
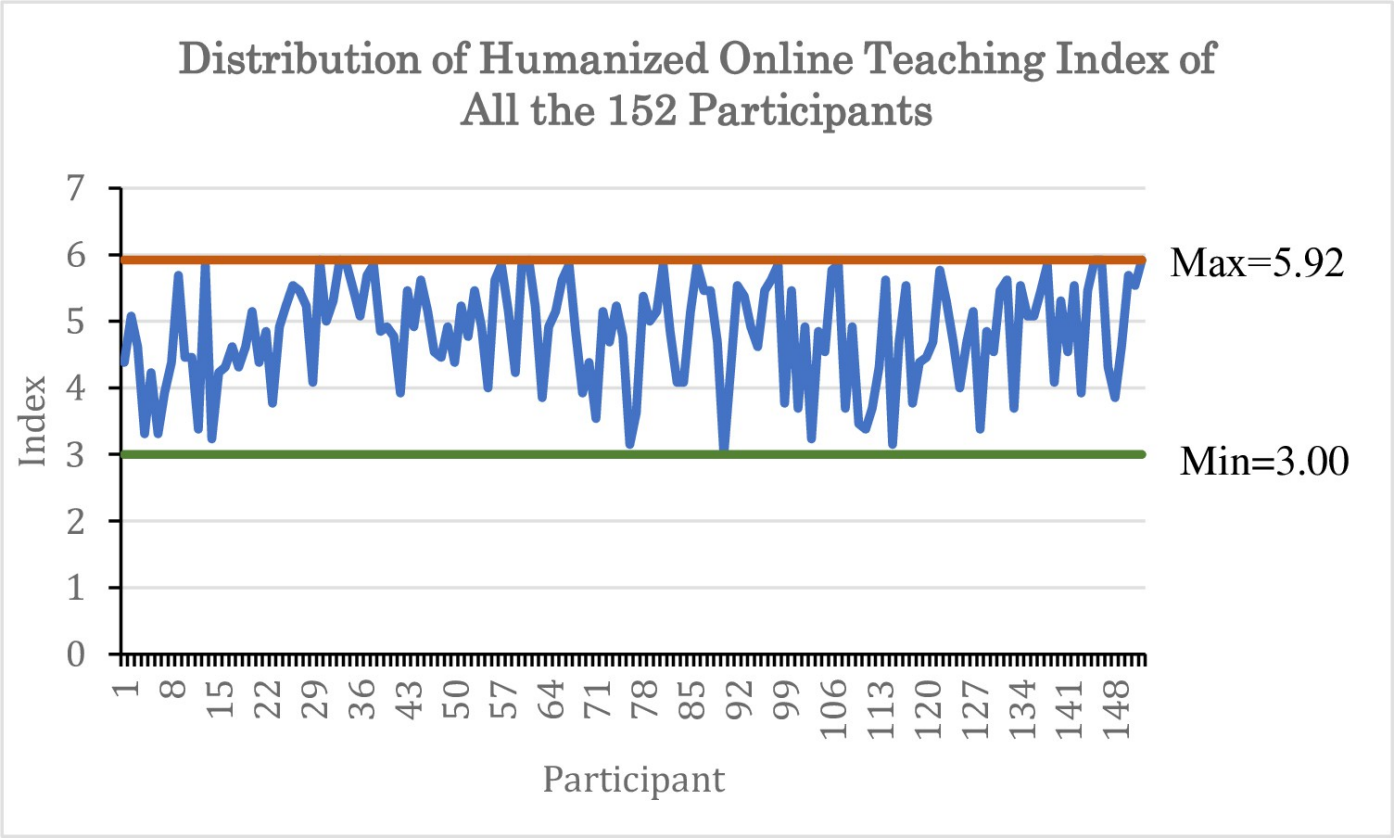
Humanized online teaching index distribution.

All 13 items appeared to be worthy of retention, resulting in a decrease in the alpha if deleted (Table 7). This demonstrates that a composite index based on these 13 items could be created to quantify the humanized online learning concept. The mean of these 13 items was computed to create a composite index, termed as Humanized Online Teaching Index, for each participant. Fig 1 illustrates the distribution of the Humanized Online Teaching index for the 152 participants in this study, revealing that the index spanned from 3.00 to 5.92.

**Table 7:** Item analysis of 13 humanized online teaching questions.

Item-Total Statistics					
	Scale Mean if Item Deleted	Scale Variance if Item Deleted	Corrected Item-Total Correlation	Squared Multiple Correlation	Cronbach’s Alpha if Item Deleted
Q16	57.45	91.057	0.357	0.196	0.774
Q17	59.21	78.525	0.445	0.294	0.769
Q18	57.36	89.703	0.257	0.196	0.784
Q19	57.82	82.743	0.490	0.397	0.761
Q20	56.82	97.317	0.186	0.134	0.784
Q21	56.91	92.574	0.358	0.215	0.775
Q22	57.86	82.968	0.445	0.340	0.766
Q23	58.16	78.968	0.569	0.362	0.751
Q24	57.85	83.295	0.478	0.370	0.762
Q25	57.58	88.245	0.432	0.268	0.768
Q26	58.54	81.442	0.531	0.353	0.756
Q27	57.53	84.701	0.491	0.302	0.761
Q28	58.42	94.524	0.301	0.198	0.779

The computed mean index of all 152 participants was 4.82, whereas the average score for the 13 items in this section was 5.92 (with Q16 to Q27 having a maximum score of 6, and Q28 having a maximum score of 5). The ratio of the mean indexes to the average score on the 13 items was 0.8138 or 81.38%. This suggests that, on average, the participants had implemented 81.38% of the humanized online teaching elements that were examined in their online instructions.

Table 8 displays the Cronbach’s alpha values that were obtained to evaluate the reliability and internal consistency of the questions that assessed the students’ online learning experiences from the instructors’ perspectives, based on the CoI framework’s social, cognitive, and teaching presences. The low alpha values obtained for social presence (0.345), cognitive presence (0.371), and teaching presence (0.300) suggest that the questions used to evaluate these presences independently were not reliable or internally consistent due to the small number of questions utilized (i.e., only three questions for each presence). The Cronbach’s alpha obtained for all nine questions was 0.436, which is less than or equal to 0.5, indicating that the consistency and internal reliability of the questions were not acceptable. Therefore, an analysis was conducted on the inter-item correlation of these items, and Q29 to Q32 were reverse-coded.

**Table 8:** Cronbach’s alphas for the three presences of the CoI framework.

Presence	Cronbach’s alpha
Social	0.345
Cognitive	0.371
Teaching	0.300

**Fig 2:**
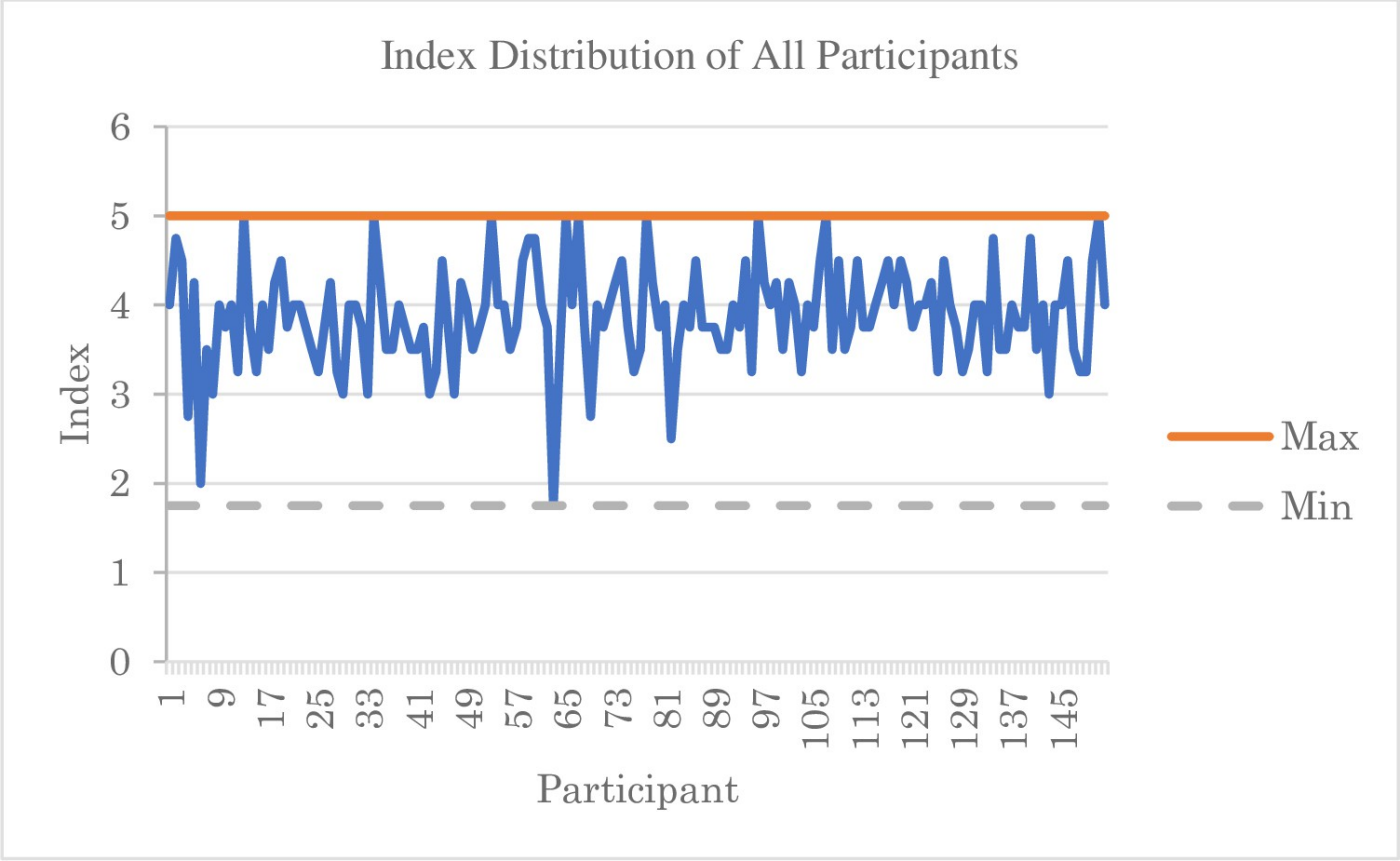
Students’ online learning experiences index distribution.

After removing items 29 to 32, the Cronbach’s alpha improved to 0.553. Deleting Q36 further raised the alpha value to 0.631 (Table 9). An alpha value ranging from 0.61 to 0.65 indicates moderate internal consistency and reliability of the items [29]. To construct an index on the teaching experiences of the instructors regarding students’ online learning experiences, Q33, Q34, Q35, and Q37 were used. The distribution of the index is shown in Fig 2, with the maximum index being 5.00 and the minimum being 1.75. The mean index obtained was 3.88. Based on the average score of 5.00 for these four items, the ratio between the mean index and average score was 0.7753 or 77.53%. These results indicate that, on average, online students’ experiences from the perspective of their teachers are good, with a rating of 77.53%.

**Table 9:** Item analysis of Q33 to Q37.

Item-Total Statistics					
	Scale Mean if Item Deleted	Scale Variance if Item Deleted	Corrected Item-Total Correlation	Squared Multiple Correlation	Cronbach’s Alpha if Item Deleted
Q33	16.15	4.222	0.327	0.137	0.492
Q34	15.33	4.858	0.415	0.256	0.460
Q35	15.45	4.514	0.499	0.334	0.411
Q36	15.51	5.205	0.093	0.010	0.631
Q37	15.77	4.139	0.353	0.162	0.473

## Discussion

### Humanized online teaching index

**Fig 3:**
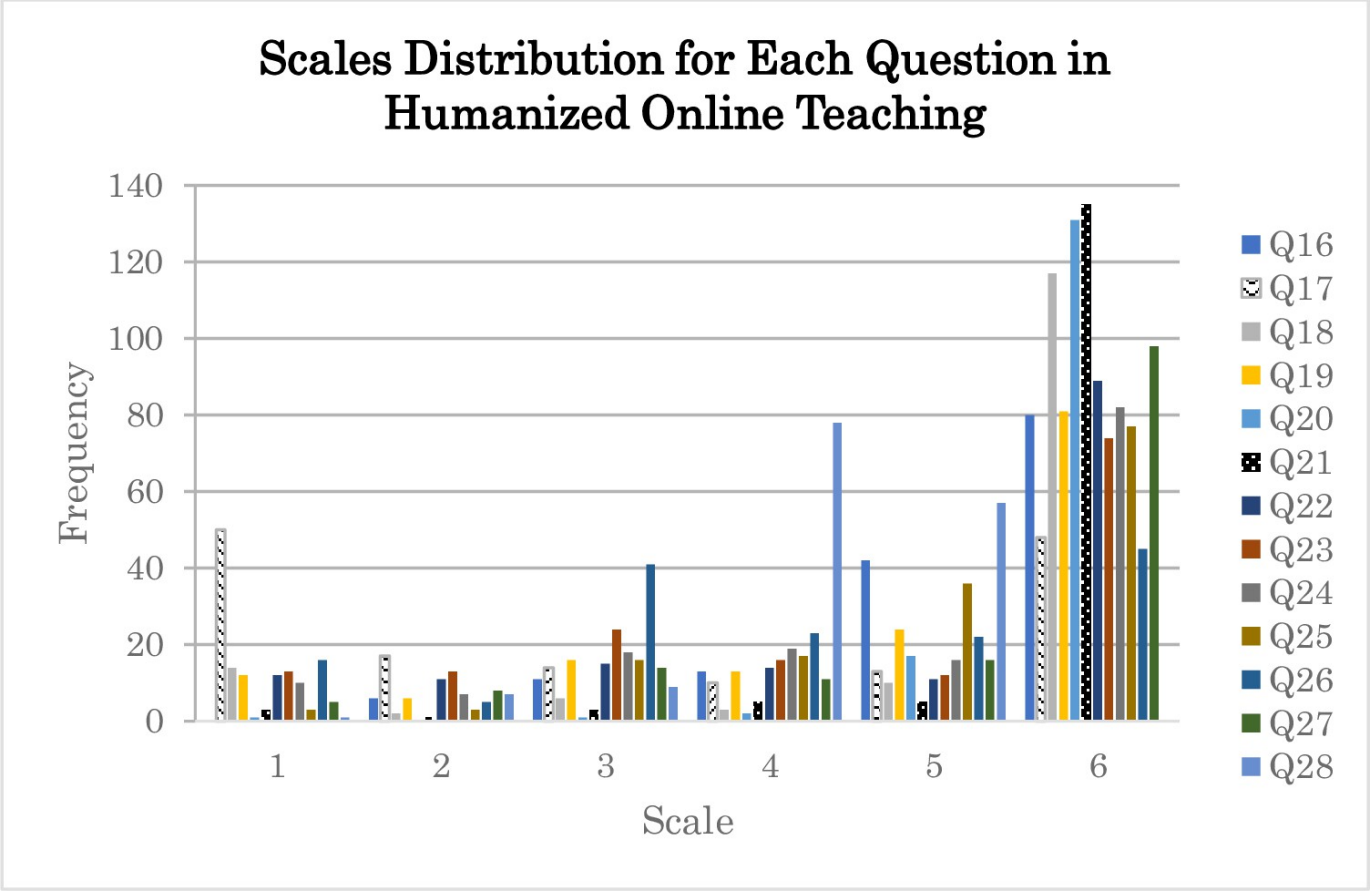
Scale distribution for Q16 to Q28 on humanized online teaching of the 152 participants.

The humanized online teaching index ranged from 3.00 to 5.92. For each of the 13 humanized online teaching items, the least frequent act is “Once a semester” for Q16 to Q27 and “Sometimes I don’t react” for Q28. Selecting the lowest frequency for all 13 items would result in an average of 1, whereas selecting the highest frequency with a score of 6 (“Almost every class”) for Q16 to Q27 and 5 (“Within the same hour”) for Q28 would achieve the maximum average of 5.92. A score of 1 would have a ratio of 0.1688 or 16.88%, meaning the participant had incorporated 16.88% of the tested humanized online teaching items in their online instructions. Based on a minimum score of 3.00 on the humanized online teaching index, we may conclude that the instructors incorporated 50.68% of the evaluated items. The derived average of 4.82 over 5.92, which is 81.38%, shows that the instructors in Malaysia had incorporated human touch in their online classes. The analysis revealed that among the tested items, Q17, Q26, and Q18 ranked highest on the lowest scale, in descending order of their scores (Fig 3). In general, participants were hesitant to display a photograph of themselves (Q17). Though they seldom monitored students who were passive in class (Q26), they always addressed students by name during online classes (Q21) and responded to their comments in chat or discussion threads to instill a feeling of individuality in them (Q20). They regularly requested students to show a photograph of themselves (Q18), but this request was often not adhered to by the instructors themselves.

### Factors contributing to humanized online teaching

In the digital age, understanding the nuances that contribute to effective and humanized online teaching is of paramount importance to not only enhancing students’ learning experience, but also fostering a more engaging and interactive virtual environment. Utilizing the devised humanized online teaching index, this study delved into the determinants that shape such practices among higher education instructors.

A multiple regression model anchored to participants’ demographic data (Table 4) and their technological preferences for online instruction (Table 5) was employed. Eight pivotal features emerged as potential influencers of humanized online teaching (Table 10).

**Table 10:** Key features influencing humanized online teaching.

No.	Question	Sig.	VIF
Q5	What subjects are you currently teaching?	p<0.001	1.567
Q6	How long have you been teaching?	p = 0.044	2.368
Q8	How good is your access to Internet at home?	p = 0.044	1.699
Q9	What type of Internet connection do you have at home?	p = 0.008	1.695
Q11_Zoom	What platform(s)/medium(s)/application(s) do you use for your online classes? •Selection on Zoom	p = 0.003	1.768
Q11_WhatsApp	What platform(s)/medium(s)/application(s) do you use for your online classes? •Selection on WhatsApp	p = 0.016	2.340
Q11_Emails	What platform(s)/medium(s)/application(s) do you use for your online classes? •Selection on Emails	p = 0.016	2.086
Q15_BModdle_After	After the COVID-19 crisis is over, which types of technology will you continue to use for face-to-face teaching in class? •Selection on Blackboard or Moodle	= 0.048	3.435

Each of these features—from the subjects taught to the platforms used—offers unique insight into the diverse facets that mold online teaching methodologies. For instance, the type of Internet connection or specific platform used for online classes might influence the fluidity and interactivity of sessions, thereby impacting the humanization aspect. Similarly, the duration of teaching experience provides insights into adaptability and evolution in teaching methods over time.

Statistically, predictors with p-values below the 0.05 threshold are deemed significant. A consistent variance inflation factor (VIF) below 10 across these features allayed concerns of multicollinearity, ensuring the reliability of our model. A deeper dive into the regression model, focusing on these eight features, is summarized in Table 11. With a multiple correlation coefficient (R) of 0.563, a moderate association emerged between the observed and predicted values of the dependent variable. The R-squared value of 0.317 suggests that our model elucidates approximately 31.7% of the variation in the dependent variable.

**Table 11:** Summary of regression model with selected features.

Parameter	Value
R	0.563
R-squared	0.317
Sig. F Change	< 0.001

The eight predictors were found to be statistically significant (with p-values less than 0.05) (Table 10). Given that the selected predictors are statistically valid, the R-squared value of 0.317 is indeed significant. As highlighted by Ozili [30], even an R-square as low as 0.1 (or 10 percent) may be deemed acceptable provided that a significant portion of the predictors are statistically valid. In our study, the selected features under consideration met this criterion, underscoring the robustness of our findings.

### Relationship between humanized online teaching and students’ online learning experience

To understand the interplay between humanized online teaching and students’ online learning experiences, we employed Pearson correlation to analyze the relationship between the constructed Humanized Online Teaching Index and the Students’ Online Learning Experiences Index. As presented in Table 12, the Pearson correlation coefficient (r) stands at 0.423, signifying a moderate positive correlation between the two indices. This suggests that an increase in the Humanized Index score corresponds to a rise in the Students’ Experiences Index score, and vice versa. The statistical significance of this correlation is underscored by a **p**-value of less than 0.001, which is significant at the 0.01 level (2-tailed). Such a result indicates that the observed correlation between the indices is highly unlikely to be a mere coincidence. Consequently, we can infer a statistically significant moderate positive relationship between the Humanized Index and the Students’ Experiences Index. In essence, this relationship suggests that a higher degree of humanization in online teaching, as gauged by the Humanized Online Teaching Index, correlates with enhanced student experiences, as captured by the Students’ Experiences Index. From a practical standpoint, this underscores the potential benefits of amplifying the human element in online teaching, as it could pave the way for enriched student experiences.

**Table 12:** Correlation between humanized index and students’ experiences index.

	Students’ Experiences Index	Humanized Online Teaching Index
Pearson Correlation (r)	0.423	
Sig. (2-tailed)	< 0.001	

## Conclusion

The study reveals that higher education instructors in Malaysia have effectively incorporated a significant human touch in their online instructions. They have integrated 81.38% of the evaluated humanized online teaching items. However, there are areas of hesitancy, such as the reluctance to display their own photograph and the monitoring of passive students. Conversely, there are positive practices where instructors consistently address students by name and respond to comments, fostering a sense of individuality among students.

Factors that are correlated with humanized online teaching include the subjects being taught, duration of teaching experience, quality of Internet access, type of Internet connection, and platforms or applications used for online classes. The regression model shows that these features are associated with approximately 31.7% of the variation in the dependent variable, indicating a potential correlation with humanized online teaching.

In terms of students’ online learning experiences, instructors with higher scores on the Humanized Online Teaching Index were associated with perceptions of better student experiences. These instructors tended to be more student-centered, creative, curiosity-driven, and interactive. This approach suggests a correlation where more humanized teaching methods are associated with perceptions of enhanced online experiences for students.

Lastly, there exists a moderate positive correlation between the Humanized Online Teaching Index and the Students’ Online Learning Experiences Index. This relationship suggests a possible correlation where higher degrees of humanization in online teaching are associated with improved perceptions of student experiences. This correlation is statistically significant, emphasizing the profound impact of integrating a human touch in online teaching on the overall student learning experience. These findings underscore the importance of humanizing online teaching, not only for the sake of the teaching process but also for the enriched learning outcomes it brings to students.

### Implications, limitations, and recommendations

This research underscores the critical role of humanizing online teaching, revealing a distinct trend: instructors who integrate more human touch elements into their teaching strategies tend to provide enhanced online learning experiences for their students. Parker, Mahler [31] support this finding, emphasizing how maximizing human interactions can mitigate students’ feelings of isolation and significantly improve their engagement and retention. These insights underscore the pressing need to incorporate more human elements in instructional methodologies, a strategy that can significantly improve the quality of student experiences. By synergizing the Humanized Online Teaching Index with the Students’ Online Learning Experiences Index, this study offers a comprehensive framework to evaluate and refine online teaching and learning practices.

Despite its contributions, this study has several limitations: 1. Reliance on Instructors’ Perspectives: The study primarily uses instructors’ perspectives to assess students’ online learning experiences, which may not fully capture the students’ actual perceptions and experiences; 2. Geographical and Contextual Constraints: The research, focused on higher education instructors in Malaysia, may have limited applicability in other geographical or educational contexts; 3. Self-Constructed Questionnaire: The absence of established measures at the time of the study led to the development of a self-constructed questionnaire. This approach could introduce biases or omit certain aspects of the human touch in online learning. Moreover, the limited number of items per construct might not sufficiently capture the full scope of the constructs, potentially affecting the robustness of the findings; and 4. Internal Consistency Measures: To enhance the reliability assessment of the constructs, future studies should consider employing additional measures of internal consistency, such as Split-Half Reliability or Average Inter-Item Correlation, alongside Cronbach’s Alpha. These methods could offer a more nuanced understanding of the questionnaire’s internal consistency, especially given the limitations of its self-constructed nature and item quantity.

Future research should aim to explore the following: 1. Solicit Student Feedback: Direct feedback from students would offer a more comprehensive view and complement the insights gained from instructors, as highlighted in [32] on the role of feedback in promoting student learning; 2. Replicate the Study in Diverse Contexts: Replicating this study in various geographical and cultural settings would enhance the generalizability of the findings, as evidenced by research such as [33] that highlights the necessity of testing hypotheses across diverse contexts to validate their external applicability and generalizability; 3. Incorporate Technological Considerations: Acknowledging the significant role of technology in online education, institutions should invest in robust technological solutions that support the human element in teaching. Jiang and Kamel Shaker Al-Shaibani [34] highlight that teaching support, learning platforms, and curriculum settings are crucial factors influencing students’ learning adaptability, especially in vocational education settings in China, underlining the necessity of comprehensive technological and curricular support systems; and 4. Continued Research and Development: As online education evolves, ongoing research is vital to identify and integrate new elements of human touch in online teaching, ensuring its continued effectiveness and relevance. This is supported by [35] who emphasizes the need for continuous research to refine online learning practices.

Educational institutions should consider providing specialized training for instructors focused on humanizing online teaching. This proactive approach can leverage the positive link between the human touch in teaching and improved student experiences, further enriching the quality of online education.
